# Cancer Care Supportive Text Messaging Program (Text4Hope) for People Living With Cancer and Their Caregivers During the COVID-19 Pandemic: Longitudinal Observational Study

**DOI:** 10.2196/53668

**Published:** 2024-04-24

**Authors:** Reham Shalaby, Wesley Vuong, Belinda Agyapong, April Gusnowski, Shireen Surood, Vincent Agyapong

**Affiliations:** 1 Department of Psychiatry University of Alberta Edmonton, AB Canada; 2 Alberta Health Services, Addiction & Mental Health, Edmonton, Canada Edmonton, AB Canada; 3 Department of Psychiatry Dalhousie University Halifax, NS Canada

**Keywords:** Text4Hope Cancer Care, COVID-19, cancer, caregivers, mental health, anxiety, depression, cancer care, Canada, Canadian, treatment, stress

## Abstract

**Background:**

Cancer is the leading cause of death in Canada, and living with cancer generates psychological demands, including depression and anxiety among cancer survivors and caregivers. Text4Hope-Cancer Care SMS text messaging–based service was provided to people with cancer and caregivers during the COVID-19 pandemic to support their mental health.

**Objective:**

The aim of this study is to examine the clinical effectiveness of and satisfaction with Text4Hope-Cancer Care in addressing mental health conditions among people living with cancer and caregivers.

**Methods:**

The study was conducted in Alberta, Canada. People who were diagnosed or receiving cancer treatment and caregivers self-subscribed to receive 3-months daily supportive cognitive behavioral therapy–based SMS text messages and a web-based survey was sent at designated time points to collect clinical and nonclinical data. The Hospital Anxiety and Depression scale (HADS) was used to examine changes in anxiety and depression symptoms after receiving the service. Satisfaction with the service was assessed using a survey with a Likert scale. Descriptive and inferential statistics were used, and test significance was considered with *P*≤.05.

**Results:**

Overall, 107 individuals subscribed to the service, and 93 completed the program (completion rate 93/107, 86.9%). A significant improvement in the anxiety symptoms (HADS-Anxiety [HADS-A] subscale) was reported after 3 months of Text4Hope-Cancer Care (*t*_11_=2.62; *P*=.02), with medium effect size (Hedges *g*=0.7), but not depression symptoms (HADS-Depression [HADS-D] subscale). Subscribers expressed high satisfaction and agreed that the service has helped them to cope with mental health symptoms and improve their quality of life. Most subscribers read the SMS text messages more than once (30/30, 100%); took time to reflect or took a beneficial action after reading the messages (27/30, 90%); and highly agreed (27/30, >80%) with the value of the received supportive SMS text messages as being relevant, succinct, affirmative, and positive. All subscribers recommended SMS text messaging for stress, anxiety, and depression and for cancer care support (30/30, 100%).

**Conclusions:**

Text4Hope-Cancer Care was well-perceived and effectively addressed anxiety symptoms among people living with cancer and caregivers during the peak of the COVID-19 pandemic. This study provides evidence-based support and insight for policy and stakeholders to implement similar convenient, economic, and accessible mental health services that support vulnerable populations during crises.

**International Registered Report Identifier (IRRID):**

RR2-10.2196/20240

## Introduction

Cancer is the leading cause of death and is responsible for 28.2% of all deaths in Canada [1]. The disease mostly affects Canadians aged 50 years and older, but it can occur at any age [1]. In 2018, 1.5 million Canadians were living with or survived cancer [1,2]. However, newly diagnosed cases are projected to increase by 40% between 2015 and 2030 [1].

Living with cancer generates ongoing psychological demands that impose a heavy toll on patients and caregivers (a person who takes care of a patient with cancer) [3]. Depression and anxiety are common mental health conditions among cancer survivors, with nearly 30% reporting clinical symptoms of depression and 40% for anxiety [4,5]. These numbers vary according to different types of cancers [4,5]. Similar results were found for caregivers, where about 1 in 2 experienced symptoms of depression and anxiety. These rates may be as high as 65% and 91% for mothers of children living with cancer [3,6].

Cancer survivors often report depression and anxiety symptoms, including the fear of the recurrence of cancer, low emotional support, social isolation, and feeling lonely or abandoned after the intensive support during the phase of the treatment [4,7-9]. Depression and anxiety symptoms negatively impact the health of cancer survivors and caregivers by eroding quality of life, decreasing treatment compliance, and increasing the risk of morbidity and mortality [4,8,10].

Several interventions have been proposed to address psychological symptoms among cancer survivors and caregivers. These interventions can include educational, cognitive, or mindfulness-based therapies which are effective in addressing mental health symptoms [11,12]. However, these interventions (eg, in-person or facilitated by trained personnel) can be costly and time-consuming when compared to mobile-based interventions (eg, internet-based cognitive behavioral therapy (iCBT) or SMS text messages) that do not require a specialist facilitator [10,13,14].

Furthermore, a major body of literature suggests that remotely delivered interventions are impactful and effective in improving mental health compared to face-to-face services [15,16]. Web-based resources have been embraced as potential solutions that can cross the likely gap between evidence-based therapies and community practice, the gap which has thrived by stigma, underrecognition of psychological symptoms and lack of trained professionals [15,17,18].

SMS text messaging has demonstrated effectiveness in the treatment and prevention of mental disorders among diverse populations [19-22]. SMS text messaging services such as Text4Support effectively reduced the risk of harm to self and other harm symptoms after 6 months of intervention in a randomized controlled trial [23] and improved distress, anxiety, and depression in clinical samples [24]. Similarly, the Text4PTSI program has achieved fidelity and significantly reduced psychological distress among public safety personnel post intervention [25,26]. SMS text message–based population-level messaging programs have reported user satisfaction rates of well over 80%, and most subscribers have reported feeling connected to support systems and improving their ability to manage anxiety, depression, and general life issues, suggesting an improvement in mental health literacy [15,27-29].

In this project, Text4Hope-Cancer Care SMS text message–based service was provided to people living with cancer and caregivers during the COVID-19 pandemic. The service aimed to support their mental health by providing a daily supportive SMS text message based on cognitive behavioral therapy (CBT) principles. The service is designed to reduce stress, anxiety, and depression symptoms associated with the pandemic among this vulnerable population. In this project, we report the results of the Text4Hope-Cancer Care program regarding its clinical effectiveness in addressing mental health conditions and the satisfaction among people living with cancer and caregivers 3 months after receiving the service.

## Methods

### Study Design and Setting

The study was conducted in Alberta, Canada, where patients diagnosed or receiving cancer treatment and caregivers were eligible to self-subscribe to receive a daily supportive SMS text message for 3 months. The messages were crafted with a CBT framework and were developed by a clinical psychologist, and then reviewed and revised by a multidisciplinary team of psychiatrists, mental health therapists, and counselors who work with patients with cancer and caregivers (Text4Hope-Cancer Care is part of the primary Text4Hope service, which was introduced to support the mental health of the general public during the COVID-19 pandemic among Albertans [30]).

Examples of the Text4Hope-Cancer Care messages are (1) do things you enjoy—these activities can remind you who you are and take your mind off cancer for a while; (2) advocate for your needs using assertiveness—assertiveness is being respectful to you and the other person. Be direct, nonaggressive, and specific; and (3) cancer affects the whole family—to help you and your family cope, try to maximize quality time together, communicate, and create a schedule together.

### Ethical Considerations

Participation in the program was voluntary, and completion of the survey did not preclude receipt of subsequent supportive SMS text messages. Subscribers could opt out of the service at any time, and no incentives or inducements for participating in the program were offered. Further details of the methodology are described in the study protocol [31]. The study was conducted according to the guidelines of the Declaration of Helsinki. Ethics approval has been granted by the University of Alberta Health Research Ethics Board (Pro00086163).

### Data Collection

Data collection was run at baseline, 6 weeks, and 3 months, and subscribers were invited to complete these surveys via a link embedded in the received SMS text message at the designated time points. Survey questions were programmed into Select Survey, a web-based survey tool [31].

**Figure 1:**
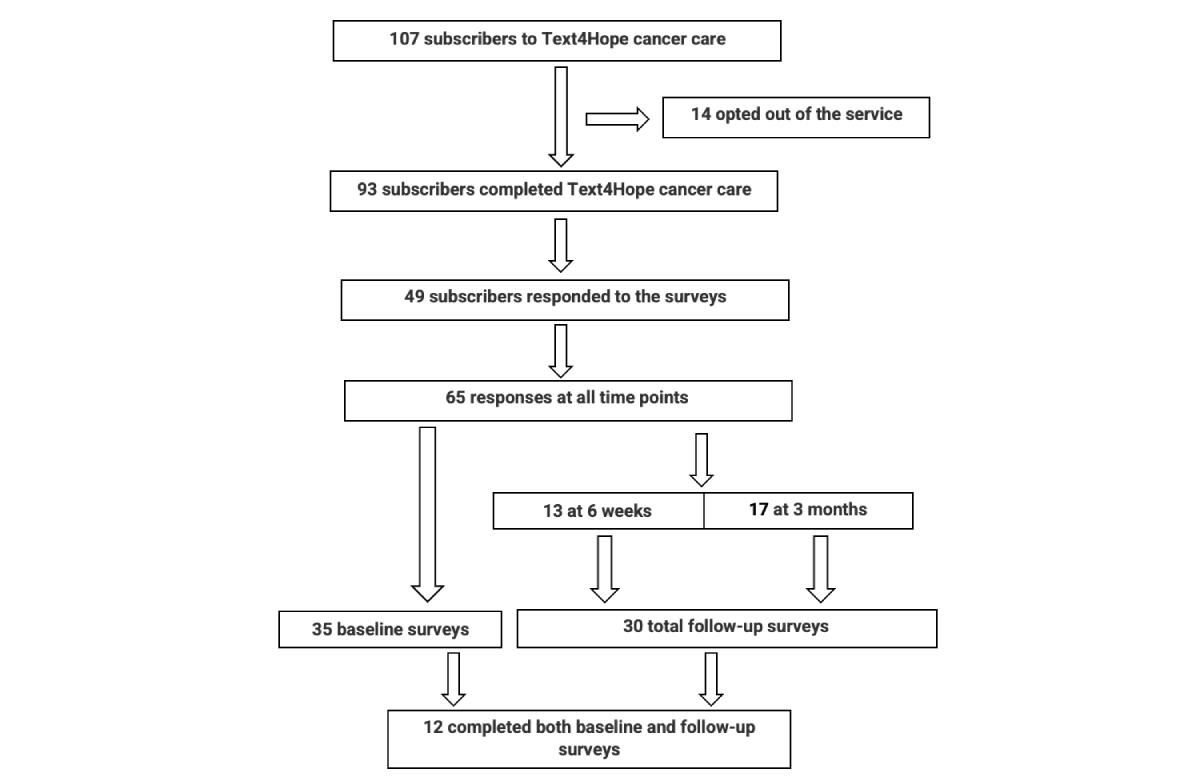
Study flowchart.

Overall, 107 subscribers participated in Text4Hope-Cancer Care program during the period between March 19, 2020, and December 19, 2022, and 14 subscribers opted out of the service (14/107, 13.1%), leaving 93 who completed the service (program completion rate of 93/107, 86.9%). From Figure 1, 49 subscribers responded to the surveys across all study time points, yielding a response rate of (49/107, 45.8%). There were a total of 65 eligible surveys received (35 at baseline and 30 at follow-up time points 6 weeks and 3 months), and only 12 subscribers have completed both the baseline and follow-up surveys. Survey questions were designed based on study objectives and available evidence from peer-reviewed literature [28,29]. The survey consisted predominantly of categorical items with Likert-scale responses and few continuously rated items. The items included sociodemographic characteristics such as gender, age, ethnic background, educational level, relationship status, employment status, and housing condition; and COVID-19–related data, such as self-isolation or quarantine due to the pandemic and perspectives regarding the likelihood of contracting COVID-19. The survey additionally included items related to cancer conditions, such as the duration of cancer diagnosis, treatment received for cancer therapy, and treatment postponed due to the COVID-19 pandemic for the participants and the loved ones who were diagnosed with cancer, if any. Clinical and service satisfaction data were also collected.

### Outcome Measures

#### Hospital Anxiety and Depression Scale

To assess the effectiveness of the Text4Hope-Cancer Care service, the Hospital Anxiety and Depression Scale (HADS) was used and presented with the 2 subscales HADS-Depression (HADS-D) and HADS-Anxiety (HADS-A) [32]. Each HADS subscale contains 7 items; each item has 4 responses with an ordinal score from 0 to 3 and a total score between 0 and 21 in each subscale, with higher scores denoting higher levels of anxious or depressive state [33]. Some items have reversed responses (ie, from 3 to 0 [3 items for HADS-D and 5 items for HADS-A]). The scale reports how participants felt and behaved during the past week [33]. Since the scale is devoid of items related to somatic symptoms or symptoms of severe psychiatric illness, it was designed to detect the common mental disorders of depression and anxiety in the adult population attending medical (nonpsychiatric) outpatient clinics [33-35]. A total score of 0-7 is considered normal, 8-10 is considered borderline abnormal (borderline case), and 11-21 is considered abnormal (case) [32].

Based on previous studies, the sensitivity and specificity of approximately (0.80) were achieved when a cutoff score of 8 or above was used to define caseness (defined as the possible and probable anxiety disorders and depression among patients in nonpsychiatric hospital clinics [32]), on both HADS-A and HADS-D subscales [34]. Cronbach α for HADS-A was 0.83 (ranging from 0.68 to 0.93), and for HADS-D was 0.82 (ranging from 0.67 to 0.90), and the correlation between the 2 subscales was 0.56 [34]. For this study, the mean scores of HADS subscales were used to detect the differences between the study groups and also to trace changes over time.

#### Satisfaction Measures

A satisfaction questionnaire was provided to collect data related to the dimensions of the study. The dimensions considered included acceptability of the service (subscriber satisfaction or experience), appropriateness (subscriber feedback related to how helpful the daily messages have been in relation to their mental health and cancer diagnosis during the COVID-19 pandemic), and subscribers’ opinion about the use of technology-based services as part of health care during crises times.

### Sample Size Calculation

Using a web-based script [36], we estimated that the sample size needed to assess the effects of the daily supportive SMS text messages among patients with cancer and caregivers on the outcome variables would be 34 with a projection that the effect size for the reduction in mean HADS-D and HADS-A scores at 3 months from baseline would be 0.2, a population variance of 1.0 for each subscale mean score, a 2-sided significance level α=.05, and a power of 80% (β=.2).

### Statistical Analysis

Descriptive statistics were performed, and chi-square or Fisher exact tests were used to define the distribution of categorical sociodemographic and cancer-related factors against 2 groups of subscribers. The first group (completers) refers to those who completed baseline and follow-up surveys, while the second group (noncompleters) refers to those who responded only to 1 survey. A post hoc analysis was run for the categorical variables with more than 2 response options when it showed a significant association, using adjusted residuals and *z* score test. A corrected *P* value was reported on this occasion (*P* value × number of comparisons). An independent 2-tailed *t* test was used to compare the continuous data of baseline mean scores of HADS subscales between the completers and noncompleters.

To examine the changes before and after receiving Text4Hope-Cancer Care SMS text messages for the completers group, a paired *t* test was used to find the difference in the mean scores of anxiety and depression HADS subscales from baseline to follow-up time points. Imputation of the missing data was applied using the Last Observation Carried Forward method, that is, the last observation (6 weeks) was carried forward (imputed) when the latest response (3 months) was missing [37]. Due to the small sample size of the completers (<20), Hedges *g* effect size was reported when the result showed significance.

For the satisfaction data, descriptive analysis was run with frequency and percentage for reporting data, using bar graphs. SPSS (version 28; IBM Corp) [38] was used to perform the statistical analysis and statistical test significance was considered with *P*≤.05.

## Results

### Text4Hope-Cancer Care Subscription Information

At baseline, subscribers were asked (multiple response questions) about their source of knowledge about the Text4Hope-Cancer Care service. The majority heard about the service from a website, such as Alberta Health Services or Mental Health Foundation websites (13/35, 37.1%), followed by social media platforms, such as Facebook and Instagram (Meta Platforms; 7/35, 20.1%). The news (eg, newspaper, web-based articles, and television), advice from a health care professional, and word of mouth (eg, family, friend, and coworker) were equally reported by the subscribers (5/35, 14.3%) each. Other sources such as employers, posters, and public libraries were also reported.

### Baseline and Sociodemographic Characteristics

Table 1 presents the results of sociodemographic, cancer-related, and clinical characteristics of the study sample. From the table, (16/48, 33.3%) of the participants were between 50 and 59 years inclusive. Participants were predominantly females (42/48, 87.5%), White (47/48, 97.9%), had attained postsecondary education (40/44, 90.9%), were in a relationship (35/44, 79.5%), had employment (25/44, 56.8%), and owned their homes (30/44, 68.2%).

Regarding COVID-19–related questions, nearly a third of the subscribers reported needing to self-isolate or quarantine during the pandemic (15/48, 31.3%). The majority were neutral regarding the likelihood that they will contract COVID-19 (16/35, 45.7%); however, compared to someone who has not been diagnosed with cancer and is not receiving treatment for cancer, half of the subscribers reported that they would more likely contract COVID-19 (9/18, 50%).

Regarding cancer-related data, there were 18 cancer cases and 17 caregivers responded to the survey at baseline. The duration of having a diagnosis of cancer was majorly split between less than 6 months, and 1 year or more duration (7/18, 38.9%), each. In terms of the received treatment for cancer, the highest proportions were reported for surgery (10/18, 55.6%) and chemotherapy (9/18, 50.0%). Having cancer treatment or surgery postponed due to the COVID-19 pandemic was reported by a few participants (3/18, 16.7%).

In terms of the questions related to the loved ones of the participants, most participants reported having had 3 or more loved ones diagnosed with cancer and received treatment for cancer (9/16, 56.3%); having a loved one passed away due to cancer (14/17, 82.4%); mostly were a partner or a parent (6/17, 35.3%, each); the loved one was diagnosed with cancer for 1 year or more (10/17, 58.8%); having had received either surgery and chemotherapy (6/17, 35.3%), each; and the majority reported having their loved ones’ cancer treatment or surgery postponed due to the COVID-19 pandemic (15/17, 88.2%).

**Table 1:** Distribution of the characteristics of the participants who responded to baseline and follow surveys (completers) and the participants who responded to only 1 survey (noncompleters).

Variable					Completers (n=12)			Noncompleters (n=37)			Total (N=49)		Chi-square (*df*)/*t* test (*df*)					*P* value		
**Age (years), n (%)**															a^a^					.12
			<40	0 (0)			10 (28)			10 (21)										
			40-49	4 (33)			5 (14)			9 (19)										
			50-59	5 (42)			11 (31)			16 (33)										
			≥60	3 (25)			10 (28)			13 (27)										
**Sex, n (%)**															a					.99
			Male	1 (8)			5 (14)			6 (13)										
			Female	11 (92)			31 (86)			42 (88)										
**Ethnicity, n (%)**															a					.99
			White	12 (100)			35 (97)			47 (98)										
			Asian	0 (0)			1 (3)			1 (2)										
**Education level, n (%)**															a					.74
			Less than high school	0 (0)			2 (6)			2 (5)										
			High school diploma	1 (8)			1 (3)			2 (5)										
			Postsecondary education	11 (92)			29 (91)			40 (91)										
**Relationship status, n (%)**															0.2 (1)					.70
			In a relationship	10 (83)			25 (78)			35 (80)										
			Not in a relationship	2 (17)			7 (22)			9 (21)										
**Employment status, n (%)**															1.5 (1)					.21
			Employed	5 (42)			20 (63)			25 (57)										
			Unemployed	7 (58)			12 (38)			19 (43)										
**Housing status, n (%)**															a					.77
			Own a home	8 (67)			22 (69)			30 (68)										
			Renting	3 (25)			5 (16)			8 (18)										
			Living with family	1 (8)			5 (16)			6 (14)										
**COVID-19 related questions, n (%)**																				
	**Self-isolate or self-quarantine due to COVID-19 symptoms, recent travel, or because being in contact with someone who may have COVID-19**													0.3 (1)					.59	
		No		9 (75)		24 (67)			33 (69)											
		Yes		3 (25)		12 (33)			15 (31)											
	**Subscribers’ perspective regarding the likelihood that they will contract COVID-19**													a					.53	
		Likely		1 (8)		6 (26)			7 (20)											
		Unlikely		5 (42)		7 (30)			12 (34)											
		Neutral		6 (50)		10 (44)			16 (46)											
	**Subscribers’ perspective regarding the likelihood that they will contract COVID-19 compared to someone who has not been diagnosed with cancer and is not receiving treatment for cancer**													a					.51	
		More likely		3 (43)		6 (55)			9 (50)											
		As likely		4 (57)		3 (27)			7 (39)											
		Less likely		0 (0)		2 (18)			2 (11)											
**Cancer-related questions reported at baseline (12 completers and 23 noncompleters)**																				
	**Type of subscribers, n (%)**													0.4 (1)					.56	
		Patients with cancer		7 (58)		11 (48)			18 (51)											
		Caregivers		5 (42)		12 (52)			17 (49)											
	**Duration of the diagnosis with cancer, n (%)**													a					.85	
		Less than 6 months ago		2 (29)		5 (46)			7 (39)											
		6 months to less than 1 year ago		2 (29)		2 (18)			4 (22)											
		1 year or more		3 (43)		4 (36)			7 (39)											
	**Cancer treatment received^b^, n (%)**																			
		Chemotherapy		6 (86)		3 (27)			9 (50)			a				.049				
		Hormone therapy		2 (29)		0 (0)			2 (11)			a				.14				
		Immunotherapy		0 (0)		0 (0)			0 (0)			N/A^c^				N/A				
		Radiation therapy		3 (43)		4 (36)			7 (39)			a				.99				
		Stem cell therapy		0 (0)		0 (0)			0 (0)			N/A				N/A				
		Surgery		4 (57)		6 (55)			10 (56)			a				.99				
		Targeted therapy		2 (29)		0 (0)			2 (11)			a				.14				
		None		0 (0)		4 (36)			4 (22)			a				.12				
	**Having cancer treatment or surgery postponed due to the COVID-19 pandemic, n (%)**													a					.99	
		Yes		1 (14)		2 (18)			3 (17)											
		No		5 (71)		8 (73)			13 (72)											
		Not applicable		1 (14)		1 (9)			2 (11)											
	**Total number of loved ones who have been diagnosed with cancer and received treatment for cancer, n (%)**													a					.55	
		1-2		2 (67)		5 (39)			7 (44)											
		≥3		1 (33)		8 (62)			9 (56)											
	**Having a loved one passed away due to cancer, n (%)**													a					.99	
		No		1 (25)		2 (15)			3 (18)											
		Yes		3 (75)		11 (85)			14 (82)											
	**Loved one diagnosed with cancer, n (%)**													a					.047	
		Partner		0 (0)		6 (46)			6 (35)											
		Parent		1 (25)		5 (39)			6 (35)											
		Friend		1 (25)		2 (15)			3 (18)											
		Child		2 (50)		0 (0)			2 (12)											
	**When was your loved one diagnosed with cancer? n (%)**													a					.41	
		Less than 6 months ago		1 (25)		5 (39)			6 (35)											
		6 months to less than 1 year ago		1 (25)		0 (0)			1 (6)											
		1 year or more		2 (50)		8 (62)			10 (59)											
	**Cancer treatment received by the loved one, n (%)**																			
		Chemotherapy		3 (60)		3 (25)			6 (35)			a				.28				
		Hormone therapy		0 (0)		3 (25)			3 (9)			a				.52				
		Immunotherapy		0 (0)		1 (8)			1 (6)			a				.99				
		Radiation therapy		2 (40)		0 (0)			2 (12)			a				.07				
		Surgery		3 (60)		2 (17)			5 (29)			a				.12				
		Targeted therapy		0 (0)		1 (8)			1 (6)			a				.99				
		Do not know		0 (0)		1 (8)			1 (6)			a				.99				
	**Loved one having cancer treatment or surgery postponed due to the COVID-19 pandemic, n (%)**													a					.99	
		Yes		4 (100)		11 (85)			15 (88)											
		No		0 (0)		2 (15)			2 (12)											
	**Baseline HADS scores (12 completers and 21 noncompleters), mean (SD)**																			
		HADS-D^d^		8.83 (5.57)		9.00 (5.35)			N/A			0.1 (31)				.93				
		HADS-A^e^		12.00 (4.90)		12.48 (4.68)			N/A			0.3 (31)				.78				

^a^a: Fisher exact test.

^b^Multiple response questions with reported relative frequency.

^c^N/A: not applicable.

^d^HADS-D: depression subscale of the Hospital Depression and Anxiety scale.

^e^HADS-A: anxiety subscale of the Hospital Depression and Anxiety scale.

Regarding group differences, only 2 questions from above showed significant differences between completers and noncompleters. A significantly higher proportion of completers reported receiving chemotherapy (6/12, 85.7%) compared to noncompleters (3/23, 27.3%; *P*=.049). Similarly, there was a significant difference regarding the loved ones who were diagnosed with cancer (*P*=.047). Post hoc analysis revealed that the proportion of the participants who had a child diagnosed with cancer who completed 2 (50%) surveys was significantly more than expected when compared to those who completed only 1 (0%) survey among the same group (*z* score=2.7; adjusted *P*=.03).

Regarding the clinical assessment, the independent *t* test revealed no significant difference between completers and noncompleters at their baseline scores of HADS-D (*t*_31_=0.09; *P*=.93) and HADS-A (*t*_31_= 0.28; *P*=.78) subscales.

### Clinical Outcome Results

Table 2 shows the changes in the mean scores of the primary outcome measure subscales, HADS-D and HADS-A, after 3 months from the baseline for participants who completed both the baseline and other follow-up surveys (completers). As illustrated in Table 2, the participants recorded lower mean scores on both HADS-D and HADS-A subscales at follow-up compared to their mean scores at baseline. However, only the HADS-A subscale was statistically significant (*t*_11_=2.62; *P*=.02), with a medium effect size (Hedges *g*=0.7).

**Table 2:** Changes of the mean scores of HADS-Da and HADS-Ab subscales after the introduction of Text4Hope cancer care.

Measure	Scores (n=12)		Change from baseline, n (%)	Mean difference (95% CI)	*P* value	*t* test (*df*)	Effect size (Hedges *g*)
	Baseline score, mean (SD)	Follow-up, mean (SD)					
HADS-D	8.83 (5.57)	7.92 (3.92)	0.91 (10.42)	0.92 (–1.32 to 3.16)	.34	0.90 (11)	0.24
HADS-A	12.00 (4.90)	9.67 (5.12)	2.33 (19.42)	2.33 (0.37 to 4.29)	.02	2.62 (11)	0.70

^a^HADS-D: depression subscale of the Hospital Depression and Anxiety scale.

^b^HADS-A: anxiety subscale of the Hospital Depression and Anxiety scale.

### Satisfaction Data

#### Overview

The feedback regarding the receptivity and satisfaction with Text4Hope cancer care service was reported by the subscribers at the follow-up time points (n=30), that is, after receiving the service.

#### Benefits of Text4Hope-Cancer Care SMS Text Messages

**Figure 2:**
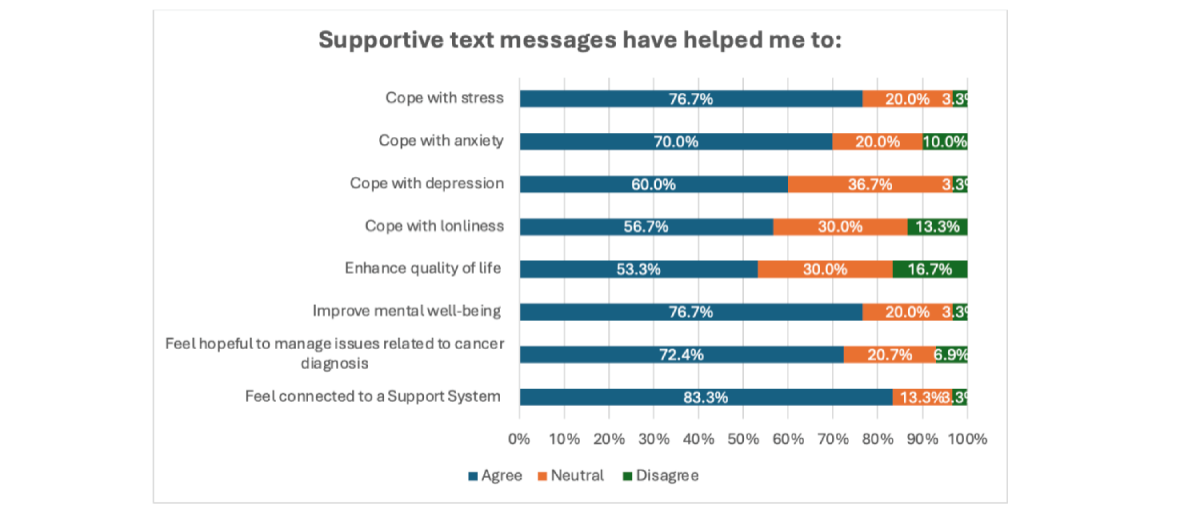
Appropriateness of Text4Hope-Cancer Care SMS text messages with patients’ mental well-being and cancer diagnosis.

Figure 2 demonstrates participants’ satisfaction measure data in relation to their perceived mental and physical health. The figure showed that the majority of the subscribers agreed that the messages have helped them to cope with symptoms related to cancer diagnosis, including stress (23/30, 76.7%), anxiety (21/30, 70%), depression (18/30, 60%), and loneliness (17/30, 56.7%). Similarly, most participants agreed that the supportive SMS text messages helped them feel connected to a support system (25/30, 83.3%), feel hopeful that they can manage issues related to cancer diagnosis (21/30, 72.4%), and that the messages improved their overall mental well-being (23/30, 76.7%). However, only 16 (53.3%) participants agreed that the messages enhanced their quality of life.

#### Receptivity of the Supportive SMS Text Messages

**Figure 3:**
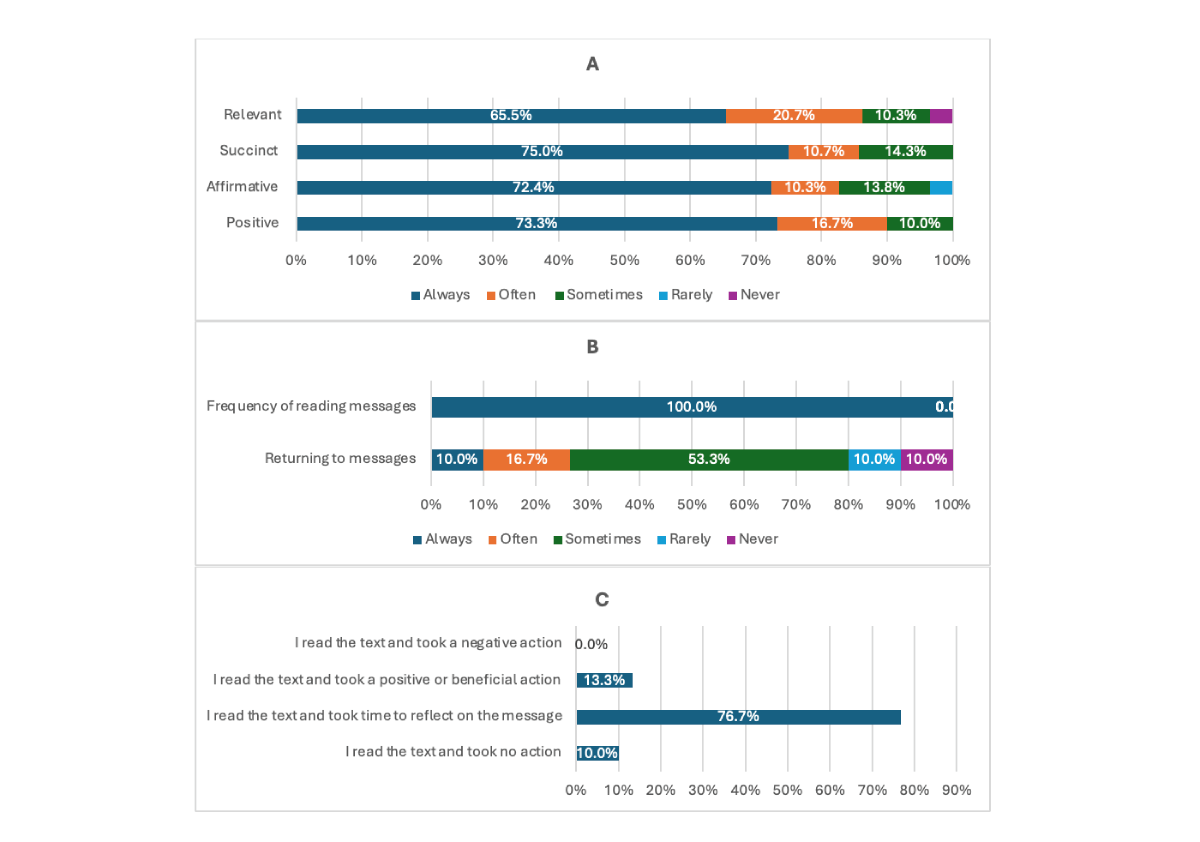
Receptivity of the supportive SMS text messages. (A) Agreement that the text messages were relevant, succinct, affirmative, and positive. (B) Reading the text messages. (C) After receiving the Text4Hope cancer care text messages.

Figure 3A demonstrates that most subscribers agreed that the SMS text messages were always or often relevant (20/29, 69%), succinct (24/28, 85.7%), affirmative (24/29, 82.8%), and positive (27/30, 90%).

In regard to reading the SMS text messages and actions taken after, Figures 3B and 3C show that all subscribers reported reading the messages (30/30, 100%). Similarly, the majority reported their return to read the messages (27/30, 90.0%), and they agreed that after reading the messages, they took time to reflect on the messages (23/30, 76.7%).

#### Overall Satisfaction With the Received Supportive SMS Text Messages and Preference of Their Schedule

**Figure 4:**
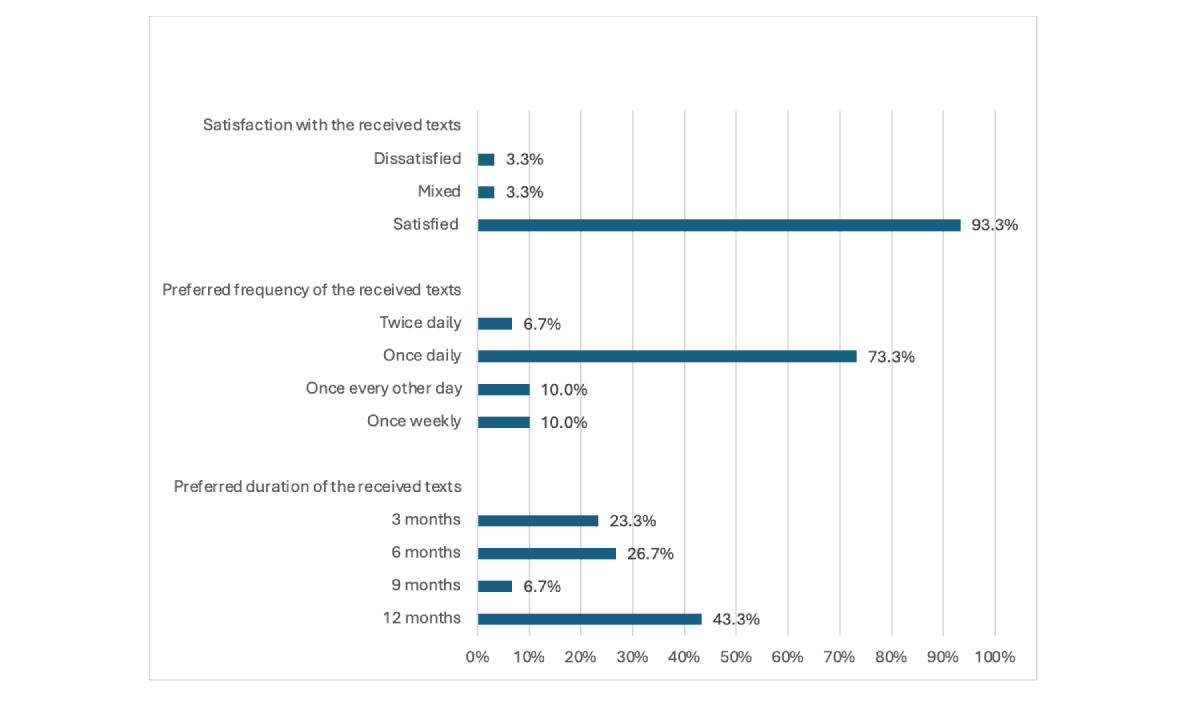
Overall satisfaction with the received supportive SMS text messages and preference of their schedule.

Figure 4 shows that most subscribers were satisfied with the service (28/30, 93.3%), preferred receiving the messages once per day (22/30, 73.3%), and the majority preferred the SMS text messages for a 12-month period (13/30, 43.3%).

#### Opinions About the Use of Technology-Based Services as Part of Health Care

**Figure 5:**
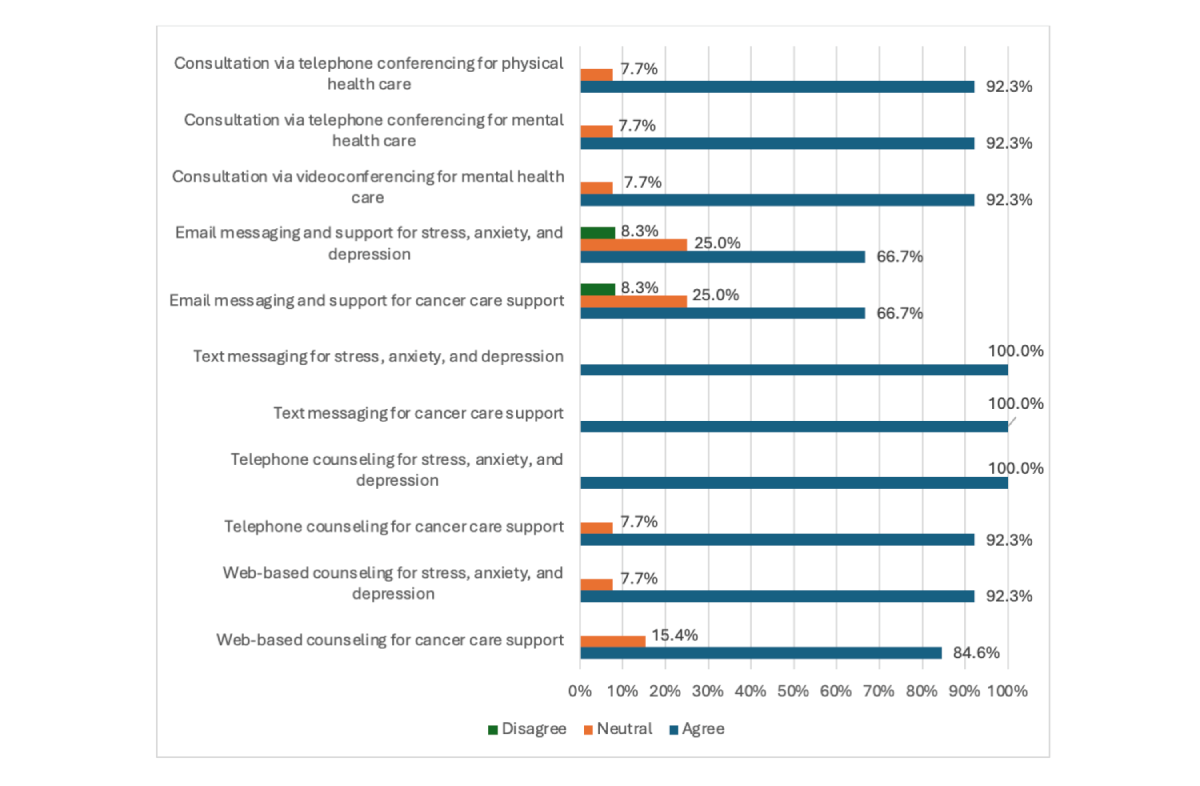
Opinions about the use of technology-based services as part of health care.

Figure 5 demonstrates that most subscribers were in agreement that everyone struggling with cancer in times of crisis, such as the COVID-19 pandemic, would recommend technology-based services as part of their health care. The top-rated recommended items were SMS text messaging for stress, anxiety, and depression (13/13, 100%); SMS text messaging for cancer care support (13/13, 100%); and telephone counseling for stress, anxiety, and depression (13/13, 100%). These were followed by consultation via telephone conferencing for physical (12/13, 92.3%) and mental health care (12/13, 92.3%); consultation via videoconferencing for mental health care (12/13, 92.3%); telephone counseling for cancer care support (12/13, 92.3%); and web-based counseling for stress, anxiety, and depression (12/13, 92.3%). Following that, web-based counseling for cancer care support was recommended by 11 (84.6%) participants. Notably, email services were relatively less preferred by the study subscribers, including email messaging and support for stress, anxiety, and depression (8/12, 66.7%), and email messaging and support for cancer care support (8/12, 66.7%).

## Discussion

### Principal Findings

This study was designed to address mental health burdens among people living with cancer and caregivers in Alberta, Canada, during the COVID-19 pandemic, using the Text4Hope-Cancer Care service. The study also examined the satisfaction of the end users with the provided service. Overall, 93 subscribers completed the Text4Hope-Cancer Care program, and 49 responded to all surveys across study time points. The principal finding of this study was the significant improvement in anxiety symptoms among the people living with cancer and caregivers who received a daily message for 3 months of Text4Hope-Cancer Care service. Although there was an observed improvement in depressive symptoms after 3 months of receiving daily supportive SMS text messages, the difference was not statistically significant.

Text4Hope cancer care represents a supportive approach provided to a vulnerable population who have been already disadvantaged by their cancer diagnosis during the pandemic time, where operations were canceled, appointments and medical care was disrupted or delayed, and hospitals were closed due to national lockdowns and pandemic obligations [3,39,40]. Other research has tapped into the need for mental health support for people living with cancer and caregivers using diverse modalities, such as cognitive‐ or mindfulness‐based, acceptance and commitment, supportive‐expressive, educational, and meaning-centered psychotherapy [11,41,42].

Our findings were not different from other SMS text messaging services provided in different contexts. Text4Support, for example, has demonstrated clinical effectiveness in reducing the risk of harm to self and other harm symptoms after 6 months of intervention in a randomized controlled trial [23,24]. The findings from this study, however, contradict findings from other studies in relation to addressing depressive symptoms. In 2 randomized controlled trials that used supportive SMS text messages, patients with depression showed symptom reduction on standardized self-report scales compared to a patient group not receiving messages (with large effect sizes: Cohen *d*=0.85 and 0.67) [21,22]. This could be explained by the heavy toll that those who live with cancer or caregivers are experiencing, particularly, during the COVID-19 pandemic. It is, however, possible, that the participants’ baseline depression would have deteriorated across the study time points without the daily supportive SMS text messages. Due to ethical considerations, this study did not include a control group who would not have received the supportive messages during the pandemic, to compare them to those who have received the service.

During the COVID-19 pandemic, people need to feel connected to a health support system. Therefore, several initiatives that included SMS text messaging services were provided and have reported comparative effectiveness to our study. Text4Hope service was provided to support the mental health of the general public in Alberta; the service reported quite similar findings to our study, where a significant reduction in stress, anxiety, depression symptoms, and suicidal ideation was achieved [14,30,43,44]. Another related service, the Text4Hope-Addiction program, was introduced to people living with substance use disorders; the service reported significant improvement in standardized measures for craving, anxiety, and depression in subscribers [45]. Similarly, the Text4PTSI program significantly reduced psychological distress among public safety personnel postintervention [25,26].

The gap in the health care service experienced by patients living with cancer seems evident and could be attributed to the lack of social and mental health support. A recent systematic review of 33 studies has highlighted the lack of social support, among others, as an exacerbating factor within the social construct for developing depression and anxiety among older patients living with cancer [9]. Such mental health symptoms, particularly depression, are linked to increased risk of mortality among people living with cancer due to psychosocial reasons such as reduced adherence to therapy and appointments; thus, the accessibility to cancer control activities and mental health support can control cancer incidence, morbidity, and mortality rates [1,9].

Our study noticed that most of the subscribers were recently diagnosed and living with cancer for less than 1 year, compared to the caregiver group, whose loved ones were living with cancer for more than 1 year. This may reflect the critical and intense periods in which these vulnerable groups may need mental health support that can span over the duration of the disease, during its early stages, as seen with people living with cancer, or later as seen with caregivers. From the literature, it was reported that the third to fifth year after diagnosis is a period that typically requires close clinical follow-up, as well as supportive care, compared to 5 years after the diagnosis, where most survivors would likely have completed their treatment [1].

Another observation in this study is that caregivers whose children were diagnosed with cancer were more committed to completing the follow-up surveys. This was supported by the fact that childhood cancer diagnosis impacts the entire family, who in turn need mental health support [46]. Aligned with this observation, a population-based cohort study in Ontario reported that the mothers of children with cancer had an increased rate of mental health outpatient visits compared to mothers in the general population [46]. This observation has persisted over decades of follow-up, reflecting the critical needs along with the commitment of the mothers of children with cancer to the provided mental health support services.

Regarding the satisfaction with the service, study subscribers expressed a high degree of satisfaction with the service in terms of coping with mental health symptoms, such as anxiety and depression and improved overall mental well-being. The subscribers highly agreed with the value of the received supportive SMS text messages, as being relevant, succinct, affirmative, and positive. These results were not different from the satisfaction levels achieved with similar services. Among the users of Text4Mood and Text4Hope, more than 3 in 4 have also endorsed an improvement in their ability to manage anxiety, depression, and general life issues, suggesting an improvement in their resilience and mental health literacy [28,29]. Several SMS text message–based population-level SMS text messaging programs have reported user satisfaction rates of well over 80% [15,27,28]. Similarly, most of Text4Mood and Text4Hope respondents reported feeling connected to support systems, as compared to our study [28,29]. In the same context, well above 50% (16/30) of the subscribers reported that the service has improved their quality of life. This parameter is of particular importance in people living with cancer who often report marked unmet survivorship care needs that consequently compromise their quality of life [8].

Participants’ responses showed satisfaction with the SMS text messages’ content and frequency. Most subscribers read the SMS text messages more than once and took time to reflect or take beneficial action after reading the messages. A similar result was obtained with Text4Hope and Text4Mood services, where more than 1 in 5 reported returning to read the SMS text messages [28,29]. Most subscribers (13/30, 43%) preferred receiving the SMS text messages for a 12-month period, as compared to lower populations who opted for 3 (23%) or 6 (27%) months. This may reflect the significant psychological burden imposed by cancer diagnosis and the ongoing need for psychological support services that give them a sense of belonging and attachment to a health care system, particularly during the tough time of the COVID-19 pandemic. Therefore, the study participants probably opted for an extended length of the service to maintain the same sense of security and belonging.

Seeing the technology services, that could be part of the health care system, support people struggling with cancer during crisis times, SMS text messaging and phone counseling were among the top-rated care services either for mental health issues or cancer-related care. This was not surprising since, nowadays, phones represent an integral part of almost everyone’s life that could facilitate communication and provide channels of support when other conventional lines are obstructed or disrupted due to crises, such as the COVID-19 pandemic. Notably, compared to phone services, email services were relatively less preferred by the study subscribers. This could be attributed to the lack of accessibility to the internet required to access emails, particularly compared to the widely spread phones and available cellular plans.

Digital technology, therefore, introduces future opportunities to support the development of a scalable mental health workforce with the potential to leverage these technologies for integrating into mental health care, particularly for people with severe mental illness and economically disadvantaged communities [19,47,48].

### Strengths and Limitations

The study is not without limitations. The low number of subscribers to the program was one of the limitations that curbed the evidence of Text4Hope cancer care to be evaluated among the rest of the community of people living with cancer and caregivers. Notwithstanding, the study reached the desired sample size as previously determined. The dropout rate of this study was aligned with other SMS text messaging services, such as Text4Hope Canada, where the dropout rate was similar (13%) and relatively higher than other web-based services (eg, iCBT) with a reported adherence rate of (52.8%) [10,43]. It is also of note that this study did not report on the time points at which the subscribers dropped out of the study (n=14); this information could have been helpful in better understanding the receptivity of the service among the targeted population and supporting the planning of future support services. Additionally, although the response rate was relatively low (49/107, 45.8%), as compared to the program completion rate (93/107, 87%), lower response rates are usually encountered with such web-based surveys, particularly when no monetary incentives were used, as the case in this study [43,49,50]. On the other hand, the relatively high response rate is one of the strengths of the study which reflects subscribers’ commitment to provide their feedback; thus, the results of the study could be generalized to all cohorts of the study.

### Conclusions

Text4Hope-Ccancer Care represents the health system and community response to close the gap during the pandemic time via providing mental health support to a vulnerable sector of the community during the pandemic time. The service was well-perceived and has successfully achieved significant effectiveness in addressing anxiety symptoms among people living with cancer and caregivers during the peak time of the COVID-19 pandemic. Compared to the costly and time-consuming conventional interventions, the SMS text messaging service represents a scalable, cost-effective intervention that can be used on any mobile technology, does not require technical skills, and does not require expensive data plans. This study provides the necessary evidence-based support and insight for policy and stakeholders to implement and guide future resource allocation to convenient, economic, and accessible mental health services that support vulnerable populations during crises.

## Abbreviations

CBT: cognitive behavioral therapy
HADS: Hospital Anxiety and Depression Scale
HADS-A: Hospital Anxiety and Depression Scale-Anxiety
HADS-D: Hospital Anxiety and Depression Scale-Depression
iCBT: internet-based cognitive behavioral therapy

## Data Availability

The data sets generated and analyzed during this study are available from the corresponding author on reasonable request.
